# Breathing pattern, accessory respiratory muscles work, and gas exchange evaluation for prediction of NIV failure in moderate-to-severe COVID-19-associated ARDS after deterioration of respiratory failure outside ICU: the COVID-NIV observational study

**DOI:** 10.1186/s12871-022-01847-7

**Published:** 2022-10-01

**Authors:** Andrey I. Yaroshetskiy, Zamira M. Merzhoeva, Natalia A. Tsareva, Natalia V. Trushenko, Galia S. Nuralieva, Vasily D. Konanykhin, Anna P. Krasnoshchekova, Sergey N. Avdeev

**Affiliations:** 1 Sechenov First Moscow State Medical University (Sechenov University), Moscow, Russia; 2 Anesthesiology and Critical Care Department, Research Institution for Clinical Surgery Division, Pirogov Russian National Research Medical University, 8/2 Trubetskaya Str., Moscow, 119991 Russia

**Keywords:** COVID-19, Noninvasive ventilation, NIV, ROX-index, Accessory respiratory muscles, Alveolar dead space, ARDS

## Abstract

**Background:**

Data on the efficacy of non-invasive ventilation (NIV) after progression of respiratory failure in patients who have already received oxygen therapy, or CPAP outside ICU is limited. The study aimed to find predictors of NIV failure based on breathing pattern, gas exchange, and accessory respiratory muscles evaluation in patients who progressed to moderate-to-severe COVID-19 ARDS.

**Methods:**

This was a prospective observational study in patients with moderate-to-severe COVID-19-ARDS on NIV (*n* = 80) admitted to COVID-ICU of Sechenov University. The combined success rate for conventional oxygen and CPAP outside ICU was 78.6% (440 of 560 patients). The primary endpoints were intubation rate and mortality. We measured respiratory rate, exhaled tidal volume (Vte), mean peak inspiratory flow (PIF), inspiratory time (Ti), PaO_2_, SpO_2_, end-tidal carbon dioxide (P_ET_CO_2_), and Patrick score, and calculated ROX index, PaO_2_/FiO_2_, ventilatory ratio, and alveolar dead space (Vdalv/Vt) on Days 1, 3, 5, 7, 10, and 14. For all significant differences between NIV success and failure groups in measured data, we performed ROC analysis.

**Results:**

NIV failure rate in ICU after deterioration of respiratory failure outside ICU was 71.3% (*n* = 57). Patients with the subsequent NIV failure were older at inclusion, more frail, had longer duration of disease before ICU admission, and higher rate of CPAP use outside ICU. ROC-analysis revealed that the following respiratory parameters after 48 h of NIV can serve as a predictors for NIV failure in moderate-to-severe COVID-19-associated ARDS: PaO_2_/FiO_2_ < 112 mmHg (AUROC 0.90 (0.93–0.97), *p* < 0.0001); P_ET_CO_2_ < 19.5 mmHg (AUROC 0.84 (0.73–0.94), *p* < 0.0001); VDalv/VT > 0.43 (AUROC 0.78 (0.68–0.90), *p* < 0.0001); ROX-index < 5.02 (AUROC 0.89 (0.81–0.97), *p* < 0.0001); Patrick score > 2 points (AUROC 0.87 (0.78–0.96), *p* = 0.006).

**Conclusion:**

In patients who progressed to moderate-to-severe COVID-19-ARDS probability of NIV success rate was about 1/3. Prediction of the NIV failure can be made after 48 h based on ROX index < 5.02, PaO_2_/FiO_2_ < 112 mmHg, P_ET_CO2 < 19.5 mmHg, and Patrick score >  = 2.

**Trial registration:**

ClinicalTrials.gov identifier: NCT04667923, registered on 16/12/2020.

**Supplementary Information:**

The online version contains supplementary material available at 10.1186/s12871-022-01847-7.

## Background

Acute respiratory failure (ARF) in COVID-19 is characterized by predominantly pulmonary dysfunction [1] and relatively low lung recruitability [2–4] that substantiate widespread use of noninvasive respiratory support methods such as constant positive airway pressure (CPAP), high-flow oxygen therapy (HFOT), and conventional oxygen therapy [5–7].

Observational studies and meta-analysis of these studies have shown high efficacy of non-invasive ventilation (NIV) in COVID-19-associated acute respiratory failure (ARF) outside the intensive care unit (ICU) [5]. The efficacy of NIV after the deterioration of respiratory failure in patients who already received conventional oxygen or CPAP outside ICU is less evident [8]. Moreover, NIV may delay tracheal intubation and increase patient self-inflicted lung injury (P-SILI), the extent of which depends on ventilatory settings, interface, and respiratory mechanics [9, 10].

Nevertheless, data on the prediction of NIV failure based on physiological respiratory parameters are limited, especially when it concerns patient respiratory drive, ventilator-derived data, alveolar dead space, and work of accessory respiratory muscles [11–13].

The study aimed to find predictors of NIV failure based on breathing pattern, gas exchange, and accessory respiratory muscles evaluation in patients who didn’t respond to the combination therapy of glucocorticoids + tocilizumab/olokizumab with conventional oxygen or CPAP outside ICU and progressed to moderate-to-severe COVID-19-ARDS.

## Methods

### Study design

This was a prospective observational clinical study (ClinicalTrials.gov NCT04667923, registered on 16/12/2020) conducted in the COVID-ICU of Sechenov University (Moscow, Russia) from October 1, 2020, to May 31, 2021. The study was approved by the Institutional Ethics Committee (reference number: 20–20, date of approval 15/07/2020). All methods were performed under the Declaration of Helsinki and the international ethical guidelines for human biomedical research. Written informed consent was waived owing to the observational nature of the study.

### Patients

Patients with COVID-19-associated acute respiratory failure receiving oxygen therapy (< 15 l/min on the non-rebreather mask) or continuous positive airway pressure (CPAP) with CPAP machines with oxygen flow < 15 l/min were daily screened for eligibility. We included screened patients with at least one of the following criteria: fatigue, excessive visible work of accessory respiratory muscles assessed by Patrick scale (4–5 points) [14], SpO_2_ < 92%. Before the entry into the study, we performed a 2-h «NIV trial» in the ICU: we switched oxygen or CPAP therapy to non-invasive ventilation using the NIV ventilator (Trilogy 202, Philips Respironics, USA) using an oro-nasal face mask for at least 2 h to assess patients’ tolerance and need for urgent intubation (CPAP 8 (8–8) cmH_2_O plus Pressure Support 10 (8–12) cm H_2_O, FiO_2_ 85 (70–100)%) to achieve the following: SpO_2_ 92–96%, exhaled tidal volume < 10 ml/kg of predicted body weight (PBW), decrease in respiratory rate, and visible work of accessory respiratory muscles. Patients were enrolled in the study if they could tolerate NIV after 2 h of NIV and didn't have the signs of deterioration (e.g., fatigue, Patrick scale 4–5 points, SpO_2_ < 92% on FiO_2_ 100%, respiratory rate > 35 per min, life-threatening heart rhythm abnormalities and/or systolic blood pressure < 80 mmHg despite norepinephrine at a dose > 2 μg/kg/min with signs of hypoperfusion, Glasgow coma score < 14 points). We used these signs of deterioration as intubation criteria for all patients throughout the study. Patients, who didn’t tolerate a 2**-**h NIV trial were urgently intubated and not included in the study. Exclusion criteria were: pregnancy, age less than 18 or more than 85 years, life-threatening heart rhythm abnormalities and/or systolic blood pressure < 80 mmHg despite norepinephrine at a dose > 2 μg/kg/min, primary lung diseases (e.g. interstitial lung diseases, lung emphysema) or tumor metastases in lungs, chronic decompensated diseases with extrapulmonary organ dysfunction (tumor progression, liver cirrhosis, congestive heart failure), Glasgow coma score < 14 points, inability to swallow, upper airways obstruction. All patients were in a self-prone position most of the time (not less than 16 h per day) [15–17], except patients with body mass index (BMI) > 35 kg/m^2^ (we placed them in lateral positions). All patients were alert or sedated in case of agitation or discomfort with a propofol infusion of 0.3–4 mg/kg/h up to the Richmond Agitation-Sedation Score (RASS) **-**1–2 points. All patients received methylprednisolone 1 mg/kg/day or dexamethasone 16 mg/day for at least 10 days, and interleukin-6(-receptor) inhibitors (tocilizumab 4 mg/kg or olokizumab 128 mg).

We did not change the PEEP level throughout the study. We corrected the pressure support level every day to achieve a minimum tolerable level (the Tobin index (respiratory rate/tidal volume) of less than 70) and Vte < 8 ml/kg IBW. NIV failure was determined as at least one of the following at preset Inspiratory pressure of 26 cmH_2_O and FiO2 100%: fatigue, Patrick scale >  = 3 points, SpO_2_ < 92%, apnoea, hemodynamic instability, or Glasgow coma score < 14 points.

### Measurements

If the patient tolerated NIV and did not have signs of deterioration after 2 h of NIV, we readjusted FiO_2_ to reach the target SpO_2_, set minimal Pressure support level to achieve Vte < 8 ml/kg IBW and Tobin index < 70, and performed the following measurements for 10 min of observation (Day 1): mean respiratory rate (RR), air leak, mean and maximum exhaled tidal volume (Vte), mean peak inspiratory flow (PIF), minimum and maximum inspiratory time (Ti), SpO_2_ with the ROX index calculation [6, 18–21], and work of accessory respiratory muscles by Patrick scale [14]. After that, we placed a mainstream capnograph between the mask and ventilatory circuit and asked the patient to make deep exhalation until the alveolar plateau was reached, and measured end-tidal carbon dioxide (P_ET_CO_2_). The final series of the survey included measurements of the respiratory pattern parameters at higher (+ 4 cmH_2_O) and lower (-4 cmH_2_O) pressure support levels—«the inspiratory pressure trial»: the mean and maximal tidal volume, mean peak inspiratory flow, mean inspiratory time, respiratory rate, and mean minute ventilation. All measurements were repeated on days 3, 5, 7, 10, and 14. After final analysis, we retrospectively calculated the HACOR score [22] at the same time points.

In patients after NIV failure, during the first 24 h after intubation, we measured plateau pressure and calculated the driving pressure at PEEP 8–10-12–14 cm of water and VT 6 ml/kg PBW, and VT + 100 ml and VT + 200 ml at PEEP 8 cmH_2_O, plot static pressure volume-curve at PEEP levels of 5 and 14 cmH_2_O (will be published elsewhere).

### Laboratory tests

After respiratory measurements, we performed arterial blood gases analysis, calculated arterial partial oxygen tension to inspiratory oxygen fraction (PaO2/FiO2) ratio, alveolar dead space (VDalv/VT), and ventilatory ratio (VR).

### Endpoints and statistical analysis

The primary endpoints were intubation rate and in-hospital mortality. Secondary endpoints included: PaO_2_/FiO_2_ ratio, ventilatory ratio, ROX index, alveolar dead space, mean and maximum expired tidal volume, maximum peak inspiratory flow, and accessory respiratory muscles workload (Patrick’s scale) on Day 1, 3, 5, 7, 10, and 14 of NIV.

Descriptive statistics included proportions for categorical and median (interquartile range) for continuous variables. No imputation was made for missing data. To assess differences between NIV success and NIV failure groups, we performed the Mann–Whitney U test for continuous variables and Chi-square or Fisher exact test for categorical variables. We performed ROC analysis for NIV failure prediction in case of significant differences between groups. We used Friedman test for variable dynamics within the group. A two-sided *p* < 0.05 was considered statistically significant. Statistical analyses were performed using SPSS Statistics version 27.0 (IBM, Armonk, NY, USA).

## Results

**Fig. 1:**
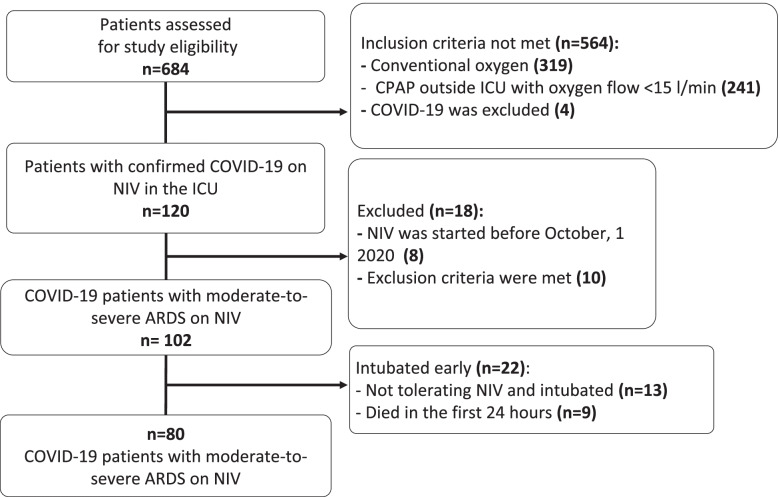
The study cohort selection

**Fig. 2:**
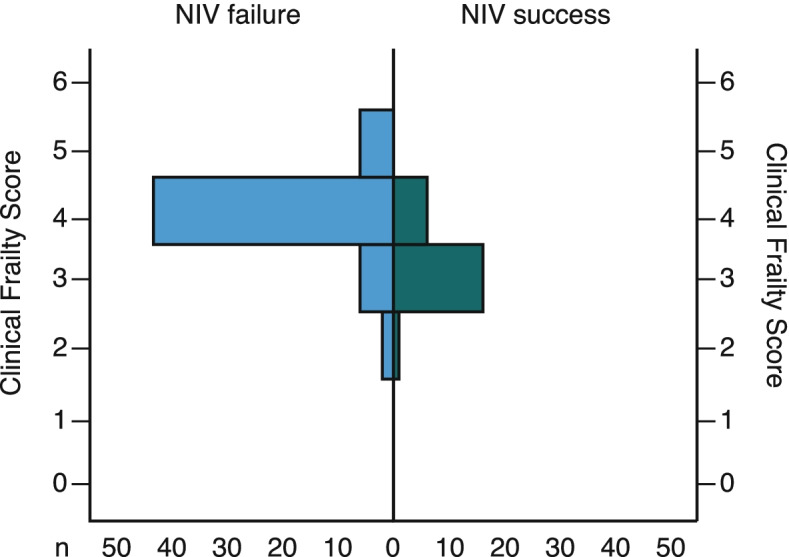
Clinical frailty score in NIV success (green) and NIV failure (blue) groups

We consecutively assessed for eligibility 684 and enrolled 80 patients (Fig. 1). Baseline demographic and laboratory characteristics, comorbidities, and medications of all patients and subgroups of NIV success, and NIV failure are summarised in Table 1. Patients with the subsequent NIV failure were older at inclusion, frailer, had a longer duration of disease before ICU admission, and higher incidence of CPAP therapy outside ICU (38.6% vs 8.7%) (Table 1). Clinical Frailty Score at inclusion is presented in Fig. 2. Overall, the combined success rate outside ICU for conventional oxygen and CPAP was 78.6% (440 of 560 patients)(Fig. 1).

**Table 1:** Patient's demographic characteristics, comorbidities, medications, and laboratory values on the first day of NIV

	**Overall** **(*n* = 80)**	**NIV success** **(*n* = 23)**	**NIV failure** **(*n* = 57)**	**p**
**Demographics**				
Age, years	71.5 [62.0–80.0]	62.0 [58.0–71.0]	73.0 [66.5–81.5]	**0.005**
Males, n (%)	54 (56.3)	12 (52.2)	33 (57.9)	0.412
BMI, kg/m^2^	30.1 [26.9–33.5]	31.1 [26.9–35.3]	30.1 [27.0–33.2]	0.404
Clinical Frailty Score, points	4 (3–4)	3 (3–4)	4 (4–4)	**0.001**
Days from disease onset, days	14.0 [9.3–18.0]	11.0 [8.0–14.0]	15.0 [10.5–20.0]	**0.005**
Days from hospital admission to ICU-NIV, days	6.0 [2.0–12.0]	4.0 [1.0–6.0]	8.5 [4.0–13.0]	**0.007**
CPAP outside ICU before ICU-NIV, n (%)	24 (30.0)	2 (8.7)	22 (38.6)	**0.008**
CPAP duration outside ICU before ICU-NIV, days	6.0 [3.3–11.0]	4.0 [3.0–4.0]	7.0 [3.8–11.5]	0.304
Comorbidities, n (%)				0.751
Hypertension	61 (76.3)	18 (78.3)	43 (75.4)	
Diabetes Mellitus	28 (35.0)	7 (30.4)	21 (36.8)	
Ischemic heart disease	20 (25.0)	6 (30.4)	14 (24.6)	
Congestive heart failure	6 (7.5)	1 (4.3)	5 (8.8)	
Atrial fibrillation	14 (17.5)	3 (13.0)	11 (19.3)	
History of stroke	5 (6.3)	2 (8.7)	3 (5.3)	
Cerebrovascular disease	12 (15.0)	3 (13.0)	9 (15.8)	
History of Cancer	5 (8.8)	1 (4.3)	4 (7.0)	
History of MI	6 (7.5)	0 (0)	6 (10.5)	
ACE inhibitors or ARB, n (%)	63 (78.8)	18 (78.3)	45 (78.9)	0.873
SOFA score	4 [3-4]	3 [2-4]	4 [3-4]	**0.001**
**Lung CT**				
Lung involvement, %	84.5 (74.0–90.0)	75.0 (70.0–86.0)	86.0 (76.5–91.5)	**0.003**
Lung consolidation, %	6.0 (4.0–8.0)	4.0 (3.0–8.0)	7.0 (5.0–8.0)	0.072
**Treatment, n (%)**				
Dexamethasone 16 mg/day or methylprednisolone 1 mg/kg/day	80 (100.0)	23 (100.0)	57 (100.0)	1.000
Enoxiparine 1 mg/kg/day	80 (100.0)	23 (100.0)	57 (100.0)	1.000
Anticytokine therapy				
Tocilizumab	67 (83.8)	19 (82.6)	45 (78.9)	0.356
Olokizumab	13 (16.2)	4 (17.4)	12 (21.1)	
**Laboratory values**				
WBC, 10^9^/l	11.2 [6.8–14.1]	9.3 [5.8–13.9]	11.7 [8.4–14.8]	0.260
Lymphocytes, 10^9^/l	0.5 [0.3–0.7]	0.7 [0.5–0.8]	0.4 [0.2–0.7]	**0.008**
D-dimer, mcg/ml	1281 [446–2147]	1070 [529–1910]	1367 [412–2593]	0.658
Fibrinogen, g/l	5.2 [3.8–7.7]	5.1 [3.5–7.0]	5.6 [4.2–7.9]	0.483
Creatinine, mcg/l	85.8 [72.7–104.5]	75.3 [68.8–89.0]	92.2 [80.4–112.1]	**0.002**
LDH, U/l	1082 [780–1537]	819 [703–1310]	1207 [875–1597]	0.116
CRP, mg/l	37.0 [12.1–92.4]	32.2 [12.3–135.7]	42.0 [11.9–92.4]	0.807

Data presented as medians [interquartile range] or n (%) where appropriate. Differences between groups Mann–Whitney U-test, Chi-square or Fisher exact test where appropriate

Lung involvement is defined as the proportion of the lung infiltrates including ground-glass opacities, crazy paving, and consolidation on high-resolution CT scan to whole lung volume. Lung consolidation is defined as the proportion of the lung consolidation volume to lung infiltrates volume

We used medications included in «Prophylaxis, Diagnostics, and Treatment of patients with COVID-19. Temporary Clinical Guideline» issued by the Russian Ministry of Health for that time (versions 5–9)

*Abbreviations*: *BMI* Body mass index, *ICU* Intensive care unit, *NIV* Noninvasive ventilation, *CPAP* Continuous positive airway pressure, *COPD* Chronic obstructive lung disease, *MI* Myocardial infarction, *ACE* Angiotensin-converting enzyme, *ARB* Angiotensin-receptor blocker, *SOFA* Sequential organ failure assessment, *CT* Computed tomography, *WBC* White blood cells, *LDH* Lactate dehydrogenase, *CRP* C-reactive protein

### Primary outcomes

Non-invasive ventilation failure rate was 71.3% (*n* = 57). All patients in the NIV failure group were intubated and mechanically ventilated, and all patients died (3 patients died on ECMO, others were not eligible for ECMO because they met exclusion criteria for ECMO, mainly age restriction (> 65 years).

### Secondary outcomes

**Fig. 3:**
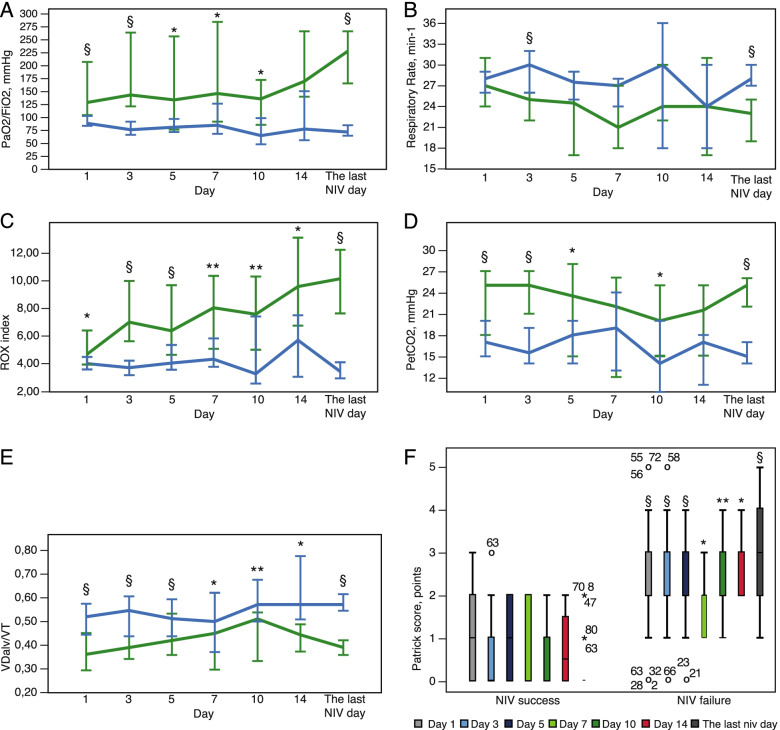
The gas exchange and respiratory pattern in NIV success and NIV failure groups during 14 days. **A** PaO_2_/FiO_2_. **B** Respiratory Rate. **C** ROX index. **D** End-tidal carbon dioxide. **E** Alveolar dead space to tidal volume ratio. **F** Patrick score. Data on NIV success (green) and NIV failure (blue) are presented as medians and 95% confidence intervals (**A**-**E**), boxplots (**F**). The x-axis represents days after initiation of non-invasive ventilation. Abbreviations: PaO_2_- partial pressure of oxygen in arterial blood; FiO_2_—inspiratory oxygen fraction; VDalv—alveolar dead space; VT- tidal volume; PetCO_2_—end-tidal partial pressure of carbon dioxide. * *p*-value < 0.05, comparison between NIV success and NIV failure groups (Mann-Whitney U test). ** *p*-value < 0.01, comparison between NIV success and NIV failure groups (Mann-Whitney U test). § *p*-value < 0.001, comparison between NIV success and NIV failure groups (Mann-Whitney U test)

Table 2 displays respiratory parameters on day 1, 3, 5, 7, 10, and 14 in all patients, NIV success and NIV failure groups. The first day of non-invasive ventilation in our study (not less than 2 h after NIV start) was associated with PaO_2_/FiO_2_ levels corresponding to moderate-to-severe ARDS (99.9 (80.0–128.5) mmHg), high alveolar dead space with hypercapnia, high ventilatory ratio, and low ROX-index (Fig. 3, Table 2). Data on Day 1 showed significant differences in widely used oxygenation and ventilation indices between NIV success and NIV failure groups, such as PaO2/FiO2, SpO2/FiO2, VDalv/VT, ROX index, and Patrick scale (Fig. 3, Table 2). But ROC analysis for these variables on Day 1 showed poor predictive value. Surprisingly, respiratory rate and ventilatory ratio being rather high, was not different at Day 1 between groups.

**Table 2:** Gas exchange and respiratory monitoring parameters in NIV success and NIV failure groups during 14 days of NIV

		**Day 1**	**Day 3**	**Day 5**	**Day 7**	**Day 10**	**Day 14**	**Last NIV day**
**Gas exchange**								
PaO_2_, mmHg	**S**	94.0** [73.0–134.0]	87.0^§^ [79.0–121.0]	76.5 [65.8–85.0]	89.0 [67.5–127.0]	74.0 [66.0–78.0]	77.0 [66.5–95.3]	84.0^§^ [75.0–107.0]
	**F**	73.0 [65.0–96.5]	66.0 [59.0–78.5]	69.5 [62.0–80.5]	69.0 [61.0–85.0]	66.0 [61.0–80.0]	67.0 [51.5–87.5]	68.0 [58.5–81.0]
FiO_2_, %	**S**	75 [50–95]*	60 [45–70]^§^	60 [34–78]^§^	60 [45–73]**	45 [43–75]**	48 [34–50]*	45 [35–50]^§^
	**F**	85 [78–100]	85 [75–100]	83 [74–100]	80 [70–95]	100 [75–100]	70 [65–93]	80 [70–95]
PaO_2_/FiO_2_, mmHg	**S**	130.8^§^ [92.0–230.0]	145.5^§^ [112.9–280.0]	135.8* [82.9–236.4]	148.3* [96.7–244.2]	137.8* [106.6–169.7]	172.2 [142.6–252.5]	231.1^§^ [162.2–280.0]
	**F**	90.0 [69.9–120.1]	77.5 [62.6–102.1]	82.4 [71.8–103.4]	86.3 [69.4–128.3]	66.0 [62.0–100.0]	78.8 [61.4–133.1]	73.0 [60.9–103.9]
SpO_2_, %	**S**	98 [96–99]^§^	97 [95–99]^§^	95 [94–97]	96 [94–98]	95 [94–98]*	96 [95–98]	97 [95–98]^§^
	**F**	95 [92–96]	93 [91–95]	95 [92–96]	95 [92–98]	93 [88–94]	94 [93–95]	94 [91–96]
SpO_2_/FiO_2_, %	**S**	128.0** [104.2–198.0]	166.7^§^ [135.7–220.0]	151.7** [115.9–233.1]	161.7** [129.8–211.1]	208.9** [132.0–225.4	203.6* [193.0–290.3]	218.9^§^ [192–256.1]
	**F**	109.4 [95.5–123.1]	109.4 [93.0–124.7]	114.0 [95.0–125.5]	118.8 [104.2–135.7]	94.0 [90.0–117.3]	135.7 [101.7–146.1]	95.0 [91.0–116.3]
RR, min-1	**S**	27.0 [24.0–33.0]	25.0^§^ [19.0–27.0]	24.5 [18.5–28.3]	21.0 [19.0–25.5]	24.0 [22.5–29.0]	24.0 [18.5–29.5]	23.0^§^ [19.0–25.0]
	**F**	28.0 [24.0–31.0]	30.0 [25.0–32.0]	27.5 [24.0–30.0]	27.0 [24.0–28.0]	30.0 [21.0–35.0]	24.0 [18.5–28.0]	28.0 [25.0–33.5]
ROX index, units	**S**	4.75* [3.80–6.62]	7.05^§^ [5.61–10.76]	6.44^§^ [4.87–9.11]	8.08** [5.33–9.72]	7.62** [5.08–9.51]	9.60* [7.03–13.21]	10.16^§^ [7.60–12.36]
	**F**	4.08 [3.42–4.96]	3.79 [2.92–4.65]	4.12 [3.57–5.52]	4.40 [3.86–5.89]	3.35 [2.67–4.56]	5.76 [4.42–6.75]	2.79 [3.52–4.63]
P_ET_CO_2_, mmHg	**S**	25.0^§^ [18.0–29.0]	25.0^§^ [21.0–27.0]	23.5* [18.0–27.3]	22.0 [15.0–25.5]	20.0* [16.5–23.5]	21.5 [16.5–24.3]	25.0^§^ [21.0–27.0]
	**F**	17.0 [14.0–21.5]	15.5 [14.0–20.7]	18.0 [12.5–20.5]	19.0 [13.0–24.0]	14.0 [11.0–18.0]	17.0 [12.0–17.5]	15.0 [13.0–18.0]
PaCO_2_, mmHg	**S**	37.0* [34.0–40.0]	39.0* [35.0–43.0]	39.0 [36.0–40.0]	37.0 [34.5–40.0]	39.0 [31.0–44.5]	37.8 [30.1–42.8]	39.0 [37.0–42.0]
	**F**	35.0 [32.0–38.0]	35.5 [33.0–38.0]	38.0 [32.0–41.3]	37.0 [37.0–39.0]	34.0 [32.0–40.0]	42.0 [29.8–51.0]	36.0 [33.0–41.0]
VDalv/VT	**S**	0.36^§^ [0.28–0.47]	0.39^§^ [0.28–0.47]	0.42 [0.38–0.52]	0.45 [0.34–0.57]	0.51 [0.38–0.53]	0.44* [0.38–0.49]	0.39^§^ [0.36–0.43]
	**F**	0.52 [0.41–0.61]	0.55 [0.41–0.64]	0.51 [0.41–0.67]	0.50 [0.37–0.62]	0.57 [0.50–0.66]	0.57 [0.53–0.69]	0.57 [0.51–0.65]
VR, units	**S**	2.91 [2.03–3.76]	2.26 [1.86–3.10]	2.48 [1.94–3.35]	2.04 [1.62–2.68]	2.58 [2.21–3.26]	2.64 [2.07–3.00]	2.13** [1.79–2.96]
	**F**	2.58 [2.17–3.26]	2.89 [2.33–3.59]	2.73 [1.74–3.05]	2.48 [2.10–3.13]	2.96 [1.91–3.55]	2.81 [1.56–3.64]	2.99 [2.33–3.88]
**Noninvasive ventilation monitoring parameters**								
Pressure support above PEEP, cmH_2_O	**S**	10.0 [7.0–10.0]	8.0* [6.0–10.0]	9.5 [7.5–11.3]	9.0 [6.0–11.0]	9.0 [4.0–12.0]	6.5 [6.0–7.8]	7.0^§^ [5.0–9.0]
	**F**	10.0 [9.0–12.0]	10.0 [8.0–12.0]	9.0 [8.0–10.3]	10.0 [8.0–12.0]	11.0 [8.0–14.0]	10.0 [7.5–10.5]	11.0 [8.0–12.0]
VTe max, ml/kg IBW	**S**	9.7 [8.2–13.4]	9.8 [8.7–11.1]	11.3 [8.6–12.9	9.3 [7.7–11.2]	10.6 [8.8–12.7]	9.6 [8.5–12.6]	10.7 [8.2–13.5]
	**F**	10.1 [8.2–12.4]	10.1 [8.0–13.2]	10.4 [8.1–13.0]	9.8 [8.2–11.3]	10.8 [8.2–14.6]	9.6 [8.5–12.6]	10.7 [8.2–13.5]
VTe mean, ml/kg IBW	**S**	7.2 [5.8–9.4]	7.6 [6.4–9.0]	7.0 [5.7–8.4]	8.1 [6.5–9.5]	8.6 [7.0–10.1]	8.4 [6.6–10.3]	7.5 [6.1–9.0]
	**F**	8.2 [6.7–10.0]	7.5 [6.6–10.2]	7.8 [7.1–11.5]	8.1 [6.3–9.0]	10.3 [7.4–13.0]	7.3 [6.9–11.7]	7.8 [6.6–11.3]
Leak mean, l/min	**S**	28 [21-40]	32 [22–43]	34 [27-40]	37 [25–50]	31 [27-34]	34 [28–44]	28 [23-39]
	**F**	30 [13-37]	30 [18-38]	27 [21-34]	29 [13–44]	32 [28-39]	30 [24–44]	32 [21-39]
PIF mean, l/min	**S**	58 [39–82]	43 [36–63]	44 [37–56]*	39 [36–49]	50 [41–56]	51 [38–73]	45 [35–56]^§^
	**F**	60 [47–86]	63 [49–85]	56 [47–85]	54 [40–69]	60 [47–79]	59 [34–63]	63 [51–92]
MV mean, l/min	**S**	17.5 [13.8–21.1]	14.1** [11.3–15.8]	12.4 [10.5–19.6]	12.6* [11.1–13.9]	16.8 [14.5–18.9]	15.3 [10.6–20.6]	13.4^§^ [10.8–15.8]
	**F**	17.2 [14.1–21.7]	19.3 [14.1–22.8]	17.0 [14.3–23.5]	15.3 [13.7–20.2]	19.6 [14.6–26.5]	17.6 [11.3–20.4]	19.4 [14.3–26.3]
Ti max, s	**S**	0.94 [0.82–1.24]	1.04** [0.92–1.36]	1.09* [0.91–1.59]	1.15 [1.00–1.24]	1.00 [0.90–1.23]	1.12 [0.82–1.56]	1.13^§^ [0.98–1.26]
	**F**	0.89 [0.79–1.06]	0.89 [0.77–1.00]	0.93 [0.78–1.05]	0.96 [0.82–1.30]	0.82 [0.73–1.20]	1.32 [1.04–1.45]	0.82 [0.70–1.02]
Ti min, s	**S**	0.76 [0.67–0.98]	0.86** [0.80–1.13]	0.92 [0.70–1.11]	1.00 [0.86–1.07]	0.79 [0.73–0.93]	0.90 [0.70–1.26]	0.93^§^ [0.72–1.08]
	**F**	0.78 [0.69–0.94]	0.76 [0.61–0.89]	0.78 [0.62–0.90]	0.90 [0.63–1.12]	0.73 [0.69–1.19]	1.13 [0.98–1.25]	0.69 [0.60–0.90]
I/E	**S**	0.71 [0.67–0.78]	0.67 [0.56–0.77]	0.71 [0.58–0.91]	0.63 [0.62–0.67]	0.69 [0.66–0.71]	0.67 [0.58–0.76]	0.67 [0.56–0.77]
	**F**	0.71 [0.59–0.77]	0.71 [0.59–0.83]	0.67 [0.58–0.77]	0.67 [0.63–0.83]	0.71 [0.63–0.83]	0.67 [0.63–0.91]	0.67 [0.59–0.77]
**Accessory respiratory muscles**								
Patrick scale, points	**S**	1.0 [0.0–2.0]^§^	0.0 [0.0–1.0]^§^	1.0 [0.0–2.0]^§^	0.0 [0.0–2.0]^*^	1.0 [0.0–1.5]^**^	0.5 [0.0–1.8]^*^	0.0 [0.0–0.0]^§^
	**F**	2.0 [2.0–3.0]	2.0 [2.0–3.0]	2.0 [1.8–3.0]	2.0 [1.0–2.0]	2.0 [2.0–3.0]	2.0 [2.0–3.5]	3.0 [2.0–4.0]
**Patients remaining on NIV**								
n (%)	**S**	23 (100.0)	23 (100.0)	14 (60.8)	9 (39.1)	5 (21.7)	4 (17.4)	23
	**F**	57 (100.0)	41 (71.9)	26 (45.6)	15 (26.3)	11 (19.3)	5 (8.8)	57
**Organ dysfunction before intubation**								
SOFA before intubation	**F**	-	-	-	-	-	-	5.0 (4.5–7.0)
Non-respiratory SOFA before intubation	**F**	-	-	-	-	-	-	1.0 (0.5–3.0)

Data presented as medians [interquartile range] or n (%) where appropriate. Differences between groups Mann–Whitney U-test, Chi-square or Fisher exact test where appropriate

*Abbreviations*: *S* Success, *F* Failure, *PaO*_*2*_ Arterial oxygen partial pressure, *FiO*_*2*_ Fraction of inspiratory oxygen, *SpO*_*2*_ Peripheral oxygen saturation, *RR* Respiratory rate, *ROX* Index SpO_2_/FiO_2_/RR, *P*_*ET*_*CO*_*2*_ End-tidal arterial carbon dioxide partial pressure, *PaCO*_*2*_ Arterial carbon dioxide partial pressure, *VDalv/VT* Alveolar dead space to tidal volume ratio, *VR* Ventilatory ratio, *PEEP* Positive end-expiratory pressure, *VTe* Exhaled tidal volume, *PIF* Peak inspiratory flow, *MV* Minute ventilation, *Ti* Inspiratory time, *I/E* Inspiratory to expiratory time ratio, *SOFA* Sequential organ failure assessment

^*^ *p*-value < 0.05, comparison between NIV success and NIV failure groups

^**^ *p*-value < 0.01, comparison between NIV success and NIV failure groups

^§^ *p*-value < 0.001, comparison between NIV success and NIV failure groups

Our data and ROC analysis showed that respiratory parameters on Day 3 (approximately 48 h after inclusion) could use as predictors of NIV failure in moderate-to-severe COVID-19-associated ARDS. Patients with NIV success showed a significant increase in PaO_2_/FiO_2_, SpO_2_/FiO_2_, ROX index, and a decrease in respiratory rate and Patrick score on Day 3, while alveolar dead space in this subgroup remained stable (Table 2, Fig. 3). On the opposite, in the NIV failure group PaO_2_/FiO_2_, SpO_2_/FiO_2_, ROX index didn’t improve, the respiratory rate even increased, and the Patrick scale showed visible work of the accessory respiratory muscles (Table 2, Fig. 3). Similar data were obtained in the retrospective analysis of the HACOR score (Supplement, Table S2, Figs. S2 and S3).

We didn’t find statistically significant differences in the respiratory pattern measured by the ventilator between NIV success, and NIV failure groups except for maximal and minimal inspiratory time on Day 3 (that were shorter in NIV failure), minute ventilation on Day 3 (that were higher in NIV failure), and peak inspiratory flow in Day 5 (that was higher in NIV failure group) (Table 2). Exhaled tidal volumes were not different between groups at all study points. Patients with NIV success showed a progressive decrease in the minute ventilation (by reducing the respiratory rate), peak inspiratory flow, and increase in inspiratory time (Table 2).

Patients within the NIV failure group had 1.0 (0.5–3.0) points of the non-respiratory SOFA on the day of intubation (Table 2). In NIV success group (*n* = 23) and in NIV failure group (*n* = 57) the duration of NIV in ICU was 6 (3–10) days vs 4 (2–8) days, respectively (*p* = 0.103). On Day 3 100% of NIV success patients remained on NIV, while in the NIV failure group it was only 71.9%, and decreased to 45.6% by the 96 h of ICU-NIV (Day 5) (Table 2).

### NIV failure prediction

**Fig. 4:**
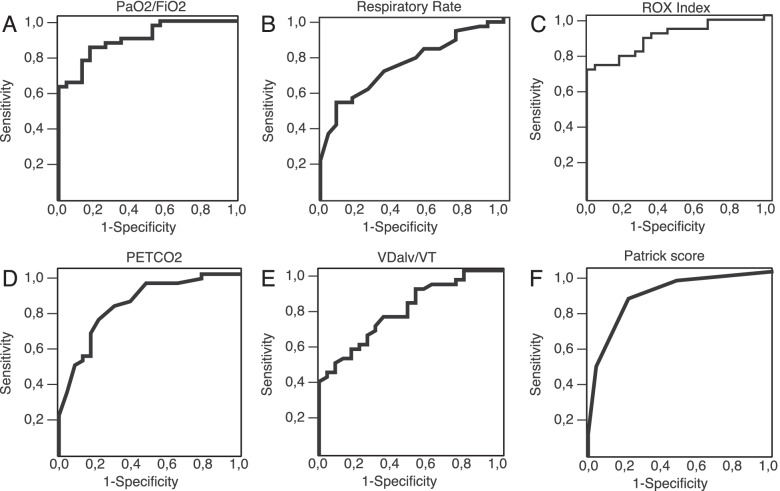
Prediction of NIV failure after 48 h basing on gas exchange and respiratory pattern parameters (ROC curves). **A** PaO_2_/FiO_2_. **B** Respiratory Rate. **C** ROX index. **D** End-tidal carbon dioxide. **E** Alveolar dead space to tidal volume ratio. **F** Patrick score. Abbreviations: PaO_2_- partial pressure of oxygen in arterial blood; FiO_2_—inspiratory oxygen fraction; VDalv—alveolar dead space; VT- tidal volume; PetCO_2_- end-tidal partial pressure of carbon dioxide

ROC analysis revealed that gas exchange parameters and accessory respiratory muscles involvement (Patrick score) after 48 h of NIV could serve as a tool for the prediction of NIV failure in moderate-to-severe COVID-19-associated ARDS: PaO_2_/FiO_2_ < 112 mmHg (Se 85%, Sp 83%, AUROC 0.90 (0.93–0.97), *p* < 0.001); P_ET_CO_2_ < 19,5 mmHg (Se 68%, Sp 83%, AUROC 0.84 (0.73–0.94), *p* < 0.001); VDalv/VT > 0.43 (Se 70%, Sp 70%, AUROC 0.78 (0.68–0.90), *p* < 0.0001); ROX-index < 5.02 (Se 78%, Sp 83%, AUROC 0.89 (0.81–0.97), *p* < 0.001); Patrick score >  = 2 points (Se 71%, Sp 90%, AUROC 0.87 (0.78–0.96), *p* = 0.006)(Fig. 4). Also, increase in the peak inspiratory flow from Day 1 to Day 3 > 4.5 l/min predicted NIV failure (Se 68%, Sp 70%, AUROC 0.72 (0.60–0.85), *p* = 0.003)(Figure S1). Data on the NIV failure prediction by retrospectively collected HACOR score presented in Supplement (Fig. S3).

**Fig. 5:**
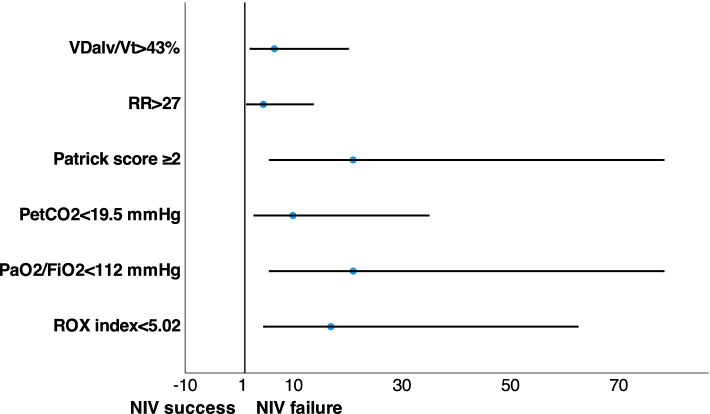
Prediction of NIV failure after 48 h basing on gas exchange and respiratory pattern parameters (Odds ratios). Data presented as odds ratio and 95% confidence interval. Abbreviations: PaO_2_- partial pressure of oxygen in arterial blood; FiO_2_—inspiratory oxygen fraction; VDalv—alveolar dead space; VT- tidal volume; PetCO_2_- end-tidal partial pressure of carbon dioxide, RR—respiratory rate

Odds ratios for these variables for NIV failure prediction were: 16.9 (4.6–62.4) 95% CI for ROX index < 5.02 (*p* < 0.001), 21.0 (5.6–78.3) 95% CI for PaO_2_/FiO_2_ < 112 mmHg (*p* < 0.001), 9.9 (2.8–35.0) 95% CI for P_ET_CO_2_ < 19.5 mmHg (*p* < 0.001), 21.0 (5.6–78.3) 95% CI for Patrick score >  = 2 (*p* < 0.003), 5.5 (2.1–20.1) 95% CI for VDalv/Vt > 43% (*p* < 0.001), and 4.4 (1.4–13.6) 95% CI for RR > 27 (*p* < 0.001) (Fig. 5).

The results of our study can be summarized as follows: 1. In patients who didn’t respond to the combination of glucocorticoids + tocilizumab/olokizumab with conventional oxygen or CPAP outside ICU, and progressed to moderate-to-severe COVID-19 ARDS, escalation of the respiratory support to noninvasive ventilation had about 1/3 probability of the NIV success. 2. The probability of NIV failure was higher in older and/or frail patients, in patients with a longer duration of COVID-19 before a NIV start, and, probably, in patients who didn’t respond to CPAP outside ICU (as compared to conventional oxygen). 3. In patients who didn’t respond to the combination of glucocorticoids + tocilizumab/olokizumab with conventional oxygen or CPAP outside ICU, progressed to moderate-to-severe COVID-19-ARDS, and escalated to NIV, prediction of the NIV failure must be made after 48 h based on respiratory physiological parameters such as the ROX index < 5.02, PaO_2_/FiO_2_ < 112 mmHg, P_ET_CO2 < 19.5 mmHg, and Patrick score >  = 2. 4. «The inspiratory pressure trial» had a low impact on the prediction of NIV failure (see Supplemental material). 5. The attempt to decrease pressure support level should be made in order to decrease lung strain if the patient has no signs of deterioration of the respiratory failure during such trial.

## Discussion

We used noninvasive ventilation as a primary tool given mono-organ lung dysfunction, relatively low lung recruitability [2–4], and high risk of severe nosocomial pneumonia in these patients due to a combination of factors, such as medical immunosuppression, high prevalence of nosocomial multidrug-resistant strains, comorbidities, and advanced age. Using NIV as a primary tool, we assumed that NIV may delay the time for tracheal intubation and increase the risk of P-SILI due to higher tidal volumes and intense respiratory efforts during NIV. The summary effect of NIV in COVID-19 may be the result of mortality reduction due to less prevalence of ventilator-associated pneumonia caused by resistant strains and mortality increase due to delayed intubation because of longer exposure to P-SILI.

Spontaneous breathing during NIV/CPAP can be harmful in patients with COVID-19-ARDS due to P-SILI, although the clinical data are limited [23, 24]. We didn’t evaluate stress and strain per se but focused on some parameters that may reflect P-SILI, such as tidal volume, peak inspiratory flow, and work of accessory respiratory muscles. Some studies in non-COVID ARF have shown that tidal volumes greater than 10 ml/kg of ideal body weight have been associated with NIV failure [25]. Moreover, Pressure Support ventilation may be more harmful than CPAP [26]. In our study, mean exhaled tidal volumes were close to 7–8 ml/kg of IBW, and didn’t differ between NIV success, and NIV failure groups as in a recently published feasibility study [13]. Pressure support levels in both subgroups of the COVID-NIV study were about 6 to 10 cmH_2_O above the PEEP level and increased during the time course in patients with subsequent NIV failure. One might assume that strain in NIV will be higher than in HFOT. The multicenter cohort trial based on data from the first wave of the COVID-19 pandemic showed increased mortality in the subgroup of NIV as compared to standard or high-flow oxygen [27]. On the contrary, randomized trials RECOVERY-RS and HENIVOT showed reduced tracheal intubation rate in the NIV group than in the HFOT group [6, 7].

Predicting NIV failure in COVID-19-ARDS by physiological variables can draw confusing results. First of all, lung involvement, distribution of the lung infiltrates, the lung recruitability, and the risk of lung overdistension can be major factors for the efficacy and the safety of noninvasive (and invasive) ventilation. Unfortunately, data concerning physiological, lung CT or other stratification before the start of any respiratory support strategy is scarce [27–31]. We found the difference in the percentage of lung involvement between NIV success and NIV failure groups, but didn’t find a significant difference in the lung CT scan picture—it was predominant bilateral diffuse ground glass opacities with a low prevalence of lung consolidation, and absence of gravity-dependent distribution of the infiltrates (neither L-, nor H-phenotype) [32]. We suggest that the greater lung involvement in the NIV failure group could lead to greater lung strain. Reanalysing data of several multicenter trials on mechanical ventilation in ARDS before the COVID-19 era, Amato et al. found that low lung recruitability could predict mortality in mechanically ventilated ARDS patients [33]. In the early days of the COVID-19 pandemic, L.Gattinoni et al. postulated low (L) and highly (H) recruitable phenotypes of COVID-19-related ARF, which could be a simple tool for stratification of respiratory support [32]. After that, several physiological studies in mechanically ventilated patients with COVID-19 [2, 3] and our recently published data from observational trial COVID-VENT [4] showed low lung recruitability in patients after NIV failure. We can hypothesize that the patients in our study had low lung recruitability, which may explain the high prevalence of NIV failure.

Second, the main tool for stratification in NIV, CPAP, and HFOT studies in COVID-19-associated ARF remains the oxygenation status (for example, PaO_2_/FiO_2_ ratio), less frequently the ventilatory status (for example, ventilatory ratio and respiratory rate), or both (for example, ROX index). But the interpretation of such stratification is often misleading. Some observational and randomized studies measured FiO_2_ during supplemental oxygenation through the face mask [6, 29] which led to overestimation of FiO_2_, underestimation of PaO_2_/FiO_2_ [34], and, as a result, overestimation of the efficacy of noninvasive respiratory support especially in severe or moderate-to-severe COVID-19-ARDS. In these studies, the application of CPAP or NIV resulted in a dramatic increase in PaO_2_/FiO_2_ during the first hours. For example, in an observational study by Coppadoro et al. [29], correct measurement of FiO_2_ during helmet-NIV demonstrated only a 22% NIV success rate in severe and about 55% in moderate COVID-19-ARDS (as compared to 50 and 83%, respectively, when FiO_2_ was measured during oxygen therapy via a face mask). In the HENIVOT randomized study [6], PaO_2_/FiO_2_ increased nearly two-fold in an hour after switching from a Venturi mask to a helmet which can be at least partially explained by the correct FiO_2_ measurement in a bi-tube ventilatory circuit of the ICU ventilator. It’s hard to distinguish the recruitment effect and correct FiO_2_ measurement after switching from low-flow oxygen to NIV/CPAP in these studies. On the opposite, in our trial, oxygenation status was assessed by online FiO_2_ measurement in the circuit during NIV in dynamics in patients with moderate-to-severe COVID-19-associated ARF, and we found that increase in this index during 48 h after the NIV start (> 112 mmHg) associated with NIV success, possibly reflecting the potential for lung recruitment in this subgroup of patients. If we make a correct comparison of the NIV success rate between the COVID-NIV study and the study by Coppadoro et al. [29] (during the helmet phase of the study, where FiO_2_ measurements were taken at the helmet inlet), we would see similar results in the moderate-to-severe COVID-19-ARDS category (28.7 vs 22.0%, respectively). Post hoc analysis of the HENIVOT study showed, that more profound stratification of patients using not only PaO_2_/FiO_2_, but in combination with dyspnoea score, and PaCO_2_ could predict the efficacy of NIV and HFNO [30]. The ROX index in our study showed a similar cut-off value for NIV failure as it was in the original study by Roca (< 4.88) [18] and COVID-19 studies using NIV [6]. Also, we focused our attention on CO_2_ removal (ventilation per se). So, we found predictive values for CO_2_ removal impairment after 48 h of NIV, such as low P_ET_CO2 (< 19.5 mmHg) and high alveolar dead space (> 43.0%), but not ventilatory ratio (that was 2–3 times normal without differences between NIV success and NIV failure groups). Our data on physiological predictors of NIV failure are in line with the study by Wendel-Garcia PD et al., where authors retrospectively investigated noninvasive respiratory support in COVID-19-associated ARF in 3 subgroups—standard oxygen, HFOT, and NIV [8]. In this study, patients in the NIV subgroup had moderate ARDS at the beginning of NIV (PaO_2_/FiO_2_ 157 [124–205] mmHg), dead space of 51, and 88% NIV failure rate. The main difference between patients’ characteristics of the COVID-NIV study and the abovementioned study was age—patients in the COVID-NIV study were older (71.5 [62–80] vs 63 [53–69]) and therefore, probably, frailer. The PaO_2_/FiO_2_ ratio in the COVID-NIV study was less than in Wendel-Garcia’s study.

Third, only several papers focused attention on accessory respiratory muscles as a predictor of the NIV failure in COVID-19-associated ARF [35–37] that were based on pilot clinical observational studies without definite cut-off values for physiological variables. In our study, an increase in peak inspiratory flow and visible (even mild) work of accessory respiratory muscles were predictors of NIV failure. Similar data were obtained in a recently published pilot study by Dargent A et al., that demonstrated the feasibility of non-invasive assessment of respiratory drive and breathing pattern during a 30-min CPAP session (negative airway pressure generated during the first 100 ms of an occluded inspiration (P0.1), tidal volume, inspiratory flow, and inspiratory time), «although it was not predictive for the intubation» [13].

The next point in the discussion of predicting NIV failure and mortality is the grade of systemic inflammatory response. We would like to mention the subclass analysis of COVID-19-ARDS performed by Sinha P et al., which demonstrated a correlation between the mortality rate and the degree of systemic inflammation [38]. In their study patients with Class 2 COVID-19-ARDS had a threefold increase in the ventilatory ratio, levels of inflammatory markers, and extremely high mortality (68, or 88%, depending on corticosteroid use) [38]. This subclass of COVID-19-ARDS resembles the patients in our study.

Also, the important factors that predicted NIV failure were age and frailty, as were shown in the Italian multicenter study of noninvasive respiratory support outside ICU WARd-COVID [31]. Our study demonstrated that elderly patients with advanced COVID-19 who didn’t respond to initial respiratory support had a higher risk of NIV failure, and in these patients, intubation and mechanical ventilation did not improve the outcome.

Data on NIV success rates outside ICU can be misleading. The meta-analysis found a very high NIV success rate outside ICU overall (about 72%), but many studies excluded patients with a ‘do-not-intubate’ (DNI) order from the analysis, who accounted for about 25%, and mortality among them reached 91% [5]. In our study, we also observed high efficiency of CPAP and low-flow oxygen outside the ICU (Fig. 1), but a high level of NIV failure when CPAP therapy outside the ICU failed. We didn’t use DNI orders (out of the law in Russia), but many patients from our cohort could be classified as DNI according to comparable studies.

One might say that in our study we procrastinated intubation. Data on the influence of timing of intubation on mortality is based on observational studies only. A systematic review and meta-analysis of these studies (*N* = 12, *n* = 8944) found no significant effect of timing of intubation on the outcome, and the duration of mechanical ventilation [39]. Moreover, in a sensitivity analysis comparing «intubation without *versus* with a prior trial of high-flow nasal cannula, or noninvasive mechanical ventilation was still not associated with a statistically detectable difference on all-cause mortality» [39]. The observational nature of these studies and the absence of predefined criteria for NIV failure made the conclusion of the meta-analysis incomplete. Tsolaki VS et al. showed us that the duration of respiratory distress (defined as PaO_2_/FiO_2_ < 100 and/or respiratory rate > 30) can lead to the progression of COVID-19-ARDS and worse outcomes [40]. In our study, we found that a long time from disease onset and hospital admission was associated with NIV failure, which could reflect more advanced disease (greater lung involvement, as seen in Table 1) and/or lower recruitability during the time course of COVID-19 (no increase in PaO_2_/FiO_2_ in NIV failure group over time, Table 2).

Early ECMO can be lifesaving in selected patients with COVID-19 [41–43]. We suggest that patients with progression of COVID-19 to moderate-to-severe ARDS without improvement in gas exchange after 48 h of NIV should be considered for ECMO. Having very high mortality after NIV failure in these patients, we can speculate that NIV failure in these patients may be one of the indications for ECMO, although they can be classified as «ECMO, no, never» with comparable mortality rate, as shown by Levy D et al. in Greater Paris [44].

To summarise our physiological considerations, we can say that the high rate of NIV failure in patients of the COVID-NIV study corresponds to other studies when we compare it with the correct measurements of physiological parameters, age, and frailty.

Our study had several limitations. First of all, it had an observational design. Second, it covered predominantly elderly patients with advanced COVID-19-associated ARF with a high risk of nosocomial infection caused by resistant strains in whom initial respiratory support (low-flow oxygen or CPAP outside ICU) failed. Third, we didn’t measure markers of P-SILI such as esophageal pressure, transpulmonary pressure, functional residual capacity, or end-expiratory lung volumes.

## Conclusions

In patients with COVID-19 who didn’t respond to the combination of glucocorticoids + tocilizumab/olokizumab with conventional oxygen (or CPAP) outside ICU, progressed to moderate-to-severe COVID-19-ARDS, and escalated to NIV, NIV success rate is about 30%. Prediction of NIV failure can be made after 48 h based on respiratory physiological parameters such as the ROX index < 5.02, PaO_2_/FiO_2_ < 112 mmHg, P_ET_CO2 < 19.5 mmHg, and Patrick score > = 2.

## Supplementary Information

**Additional file 1. **

## Data Availability

The datasets used and/or analysed during the current study are available from the corresponding author on reasonable request.

## Abbreviations

ARDS: Acute respiratory distress syndrome
ARF: Acute respiratory failure
AUROC: Area under receiver operator curve
BMI: Body mass index
CPAP: Continuous positive airway pressure
ECMO: Extracorporeal merman oxygenation
FiO_2_: Inspiratory fraction of oxygen
HFOT: High-flow oxygen therapy
IBW: Ideal body weight
ICU: Intensive care unit
NIV: Noninvasive ventilation
P0.1: Negative airway pressure generated during the first 100 ms of an occluded inspiration
PaCO_2_: Arterial carbon dioxide tension
PaO_2_: Partial pressure of oxygen in arterial blood
P_ET_CO_2_: End-expiratory carbon dioxide tension
PIF: Peak inspiratory flow
P-SILI: Patient self-inflicted lung injury
RASS: Richmond Agitation-Sedation Score
ROC: Receiver operator curve
RR: Respiratory rate
SpO_2_: Peripheral oxygen saturation
Ti: Inspiratory time
VDalv/VT: Alveolar dead space
VR: Ventilatory ratio
VT: Tidal volume
Vte: Exhaled tidal volume
