# Supplementary material for: Breathing pattern, accessory respiratory muscles work, and gas exchange evaluation for prediction of NIV failure in moderate-to-severe COVID-19-associated ARDS after deterioration of respiratory failure outside ICU: the COVID-NIV observational study
Source: BMC Anesthesiol. 2022 Oct 1;22:307. doi: 10.1186/s12871-022-01847-7 (PMC9525938; doi:10.1186/s12871-022-01847-7)
Supplement: Supplementary file 1 — Additional file 1. [file 12871_2022_1847_MOESM1_ESM.docx]

**Online Data Supplement
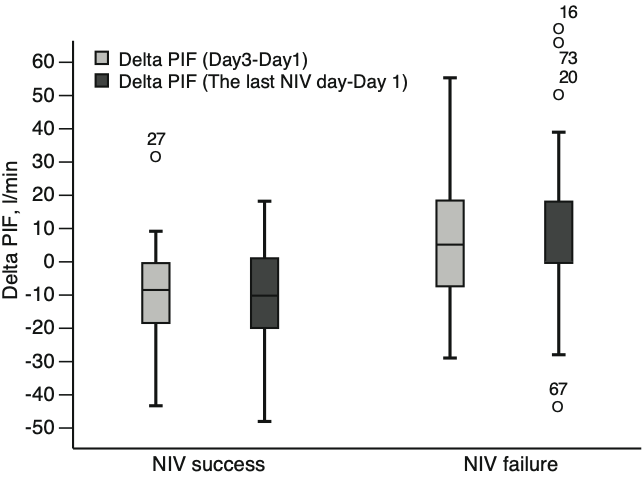
**

**Figure S1. Change in the peak inspiratory flow between the first and the 3rd, and between the first and the last NIV day in NIV success and NIV failure groups.**

Data are presented as medians and 95% confidence intervals. Grey colour depicts difference in the peak inspiratory flow during the first 48 hours of NIV, black colour - between the first and the last day of NIV.

* p-value < 0.05, comparison within subgroup of NIV success and NIV failure (Friedman test);

** p-value < 0.01, comparison within subgroup of NIV success and NIV failure (Friedman test);

§ p-value < 0.001, comparison within subgroup of NIV success and NIV failure (Friedman test).

**Respiratory pattern after pressure support level change**

The respiratory pattern response to the increased pressure support level in the NIV success group revealed the following significant changes: increase in maximal exhaled tidal volume on day 3 and 5; increase in mean exhaled tidal volume on day 1, 3, and 5; increase in peak inspiratory flow on day 1 and 3 (**Table S1**). Decreased pressure support levels in these patients showed decrease in maximum exhaled tidal volume on day 1 and 3, a decrease in mean exhaled tidal volume on day 1 and 3, and a decrease in peak inspiratory flow on day 1 (**Table S1**). Similar changes were noted in the NIV failure group (**Table S1**). There were no statistically significant changes in respiratory rate and inspiratory time in either group during pressure support level change.

**Table S1. Changes in respiratory pattern after pressure support level increase and decrease**

|  |  |  | **Day 1** | **Day 3** | **Day 5** | **Day 7** | **Day 10** | **Day 14** | **The last NIV day** |
| --- | --- | --- | --- | --- | --- | --- | --- | --- | --- |
| **Noninvasive ventilation monitoring parameters** | | | | | | | | |  |
| PS, cmH_2_O | **PS** | **S** | 10.0 [7.0-10.0] | 8.0 [6.0-10.0] | 9.5 [7.5-11.3] | 9.0  [6.0-11.0] | 9.0  [4.0-12.0] | 6.5  [6.0-7.5] | 7.0  [5.0-9.0] |
|  |  | **F** | 10.0 [9.0-12.0] | 10.0 [8.0-12.0] | 9.0 [8.0-10.3] | 10.0  [8.0-12.0] | 11.0  [8.0-14.0] | 10.0  [7.5-10.5] | 11.0  [8.0-12.0] |
|  | **PS+4** | **S** | 13.0^§^ [10.0-14.0] | 13.0^§^ [10.0-14.0] | 13.5** [11.5-15.3] | 13.0* [10.0-15.0] | 14.5 [12.3-16.0] | 10.5*  [10.0-11.8] | 11.0  [9.8-13.3] |
|  |  | **F** | 14.0^§^ [13.0-16.0] | 14.0^§^ [12.0-16.0] | 13.0^§^ [12.0-14.5] | 14.0** [12.0-16.0] | 15.0* [12.0-19.0] | 14.0 [11.5-14.5] | 15.0^§^ [12.0-16.0] |
|  | **PS**  **-4** | **S** | 6.0**  [4.0-7.0] | 4.5^§^  [2.0-6.0] | 6.0**  [4.0-7.5] | 5.0*  [2.0-7.0] | 6.5  [4.3-8.0] | 3.5  [2.3-4.0] | 4.0  [2.0-5.8] |
|  |  | **F** | 6.0^§^  [5.0-8.0] | 6.0^§^  [4.0-8.0] | 5.0^§^  [4.0-6.5] | 6.0**  [4.0-8.0] | 7.0*  [4.0-11.0] | 6.0  [3.5-6.5] | 7.0^§^  [5.0-8.0] |
| VTe max, ml/kg IBW | **PS** | **S** | 9.7  [8.2-13.5] | 9.8 [8.7-11.1] | 11.3 [8.6-12.9] | 9.4  [7.7-11.2] | 10.6  [8.8-12.7] | 11.4 [9.3-13.1] | 9.7  [8.7-11.5] |
|  |  | **F** | 10.1  [8.3-12.4] | 10.9  [8.9-13.2] | 10.4 [8.1-13.0] | 9.8  [8.2-11.3] | 10.8  [8.2-14.6] | 9.6  [8.5-12.6] | 10.7  [8.2-13.5] |
|  | **PS+4** | **S** | 10.1  [8.9-13.4] | 10.9** [10.1-13.3] | 11.9* [10.4-14.2] | 11.0 [9.3-12.1] | 11.0 [10.9-11.8] | 12.4 [10.0-14.0] | 10.5*  [10.0-13.0] |
|  |  | **F** | 11.6^§^ [9.5-13.7] | 10.8^§^  [8.9-14.1] | 11.5** [9.6-14.0] | 10.4  [9.8-12.2] | 12.6 [9.6-16.7] | 11.9 [9.1-15.3] | 11.9^§^ [9.0-14.5] |
|  | **PS**  **-4** | **S** | 8.2**  [6.3-11.0] | 9.1* [7.5-10.4] | 9.5 [7.9-11.1] | 8.8  [8.1-10.8] | 8.9  [8.0-11.0] | 9.3  [6.6-11.7] | 8.2*  [6.8-9.5] |
|  |  | **F** | 9.4  [7.6-11.0] | 9.3*  [7.7-10.7] | 10.2 [8.4-11.3] | 9.0  [7.7-11.5] | 11.8 [8.3-14.7] | 8.7  [7.6-12.1] | 9.6**  [7.4-12.0] |
| VTe mean, ml/kg IBW | **PS** | **S** | 7.2  [5.8-9.4] | 7.6  [6.4-9.0] | 7.0  [5.7-8.4] | 8.1  [6.2-9.5] | 8.6  [7.0-10.1] | 8.4  [6.6-10.3] | 7.5  [6.1-9.0] |
|  |  | **F** | 8.2  [6.7-10.0] | 7.5  [6.6-10.2] | 7.8  [7.0-11.5] | 8.1  [6.3-9.0] | 10.3  [7.4-13.0] | 7.3  [6.9-11.7] | 7.8  [6.6-11.3] |
|  | **PS+4** | **S** | 8.3*  [6.2-9.8] | 8.5**  [8.0-10.5] | 9.3*  [7.8-10.8] | 9.0  [7.8-10.1] | 9.9  [6.9-10.5] | 9.7  [7.5-10.4] | 8.2  [7.2-9.4] |
|  |  | **F** | 9.4^§^  [6.9-11.3] | 8.1*  [6.9-12.5] | 9.0*  [7.8-11.7] | 8.7  [7.2-10.0] | 10.8  [7.6-13.2] | 8.7  [7.5-11.9] | 8.6**  [7.0-11.9] |
|  | **PS**  **-4** | **S** | 6.2*  [5.0-8.3] | 6.8^§^  [5.2-7.9] | 7.4  [6.6-9.3] | 6.8  [5.7-7.4] | 7.4  [6.2-8.6] | 7.3  [5.2-9.6] | 6.3*  [5.2-7.9] |
|  |  | **F** | 7.5**  [6.2-9.6] | 7.0*  [6.0-9.1] | 7.4  [6.2-9.9] | 8.1  [6.8-9.3] | 8.8  [6.5-11.2] | 7.1  [6.5-11.6] | 7.7*  [6.1-10.4] |
| PIF mean, l/min | **PS** | **S** | 57.9 [39.0-82.0] | 43.0 [36.0-63.0] | 43.5 [36.8-56.0] | 38.7  [35.5-48.9] | 49.5 [41.3-56.3] | 50.5 [38.2-71.3] | 44.8 [34.5-55.5] |
|  |  | **F** | 60.0 [47.1-85.5] | 63.0 [49.0-84.5] | 56.5 [46.5-84.5] | 54.0 [40.0-69.0] | 59.8 [47.0-79.0] | 59.0 [33.5-62.5] | 62.5 [51.3-91.5] |
|  | **PS+4** | **S** | 60.6* [45.0-90.0] | 53.0** [44.0-68.5] | 51.0 [39.8-63.3] | 49.0 [40.4-55.0] | 50.7 [20.5-70.7] | 59.0 [42.6-76.8] | 48.0^**^ [39.6-60.3] |
|  |  | **F** | 71.2* [53.3-92.3] | 71.5^§^ [53.5-96.5] | 73.0^§^ [53.0-100.0] | 57.5 [42.5-83.3] | 81.0 [56.0-98.0] | 63.0 [41.0-67.5] | 75.0^§^ [63.0-105.0] |
|  | **PS**  **-4** | **S** | 44.0** [32.0-62.0] | 41.5 [33.5-56.3] | 48.0 [32.5-66.9] | 42.0 [36.0-45.5] | 57.5 [36.4-66.3] | 42.0 [30.6-53.8] | 42.0^*^ [29.0-53.8] |
|  |  | **F** | 55.0^§^ [43.3-68.0] | 63.0 [47.5-77.0] | 64.0 [42.0-72.0] | 44.0 [37.8-64.8] | 53.3 [44.0-81.0] | 50.0 [34.0-62.0] | 62.2* [45.8-85.0] |
| RR, min^-1^ | **PS** | **S** | 27 [24-33] | 25 [19-27] | 25 [19-28] | 21 [19-26] | 24 [22-29] | 24 [19-30] | 23 [19-25] |
|  |  | **F** | 28 [24-31] | 30 [25-32] | 28 [24-30] | 27 [24-28] | 30 [21-35] | 24 [19-28] | 28 [25-34] |
|  | **PS+4** | **S** | 26 [24-31] | 23 [19-28] | 23 [19-27]* | 24 [21-28] | 26 [20-31] | 26 [20-29] | 24 [19-27] |
|  |  | **F** | 28 [23-31] | 30 [25-32] | 28 [24-30] | 25 [22-27] | 29 [24-32] | 22 [20-26] | 29 [25-33] |
|  | **PS**  **-4** | **S** | 28 [26-31] | 25 [21-28] | 23 [20-28]* | 23 [20-29] | 27 [23-32] | 26 [21-28] | 22 [19-27] |
|  |  | **F** | 27 [25-32] | 29 [24-34] | 28 [24-33] | 25 [22-27] | 27 [20-34] | 22 [17-28] | 30 [24-33] |
| Ti max, s | **PS** | **S** | 0.94 [0.82-1.24] | 1.04  [0.92-1.36] | 1.09  [0.91-1.59] | 1.15 [1.00-1.24] | 1.00 [0.90-1.23] | 1.12 [0.82-1.56] | 1.13 [0.98-1.26] |
|  |  | **F** | 0.89 [0.79-1.06] | 0.89 [0.77-1.00] | 0.93 [0.78-1.05] | 0.96 [0.82-1.30] | 0.82 [0.73-1.20] | 1.32 [1.04-1.45] | 0.82 [0.70-1.02] |
|  | **PS+4** | **S** | 0.88 [0.81-1.22] | 1.01 [0.89-1.21] | 1.24 [0.95-1.75] | 1.13 [1.10-1.20] | 1.03 [0.88-1.36] | 0.95 [0.88-1.38] | 1.10 [0.93-1.35] |
|  |  | **F** | 0.93 [0.78-1.10] | 0.82 [0.71-0.97] | 0.96 [0.84-1.09] | 1.04 [0.80-1.14] | 0.88 [0.82-1.09] | 1.09 [0.98-1.54] | 0.82 [0.68-1.08] |
|  | **PS**  **-4** | **S** | 0.97 [0.83-1.10] | 1.02 [0.88-1.23] | 1.14 [0.93-1.36] | 1.10 [1.00-1.25] | 1.10 [0.89-1.29] | 1.05 [1.01-1.29] | 1.09 [0.95-1.26] |
|  |  | **F** | 0.90 [0.76-1.10] | 0.90 [0.77-1.02] | 0.96 [0.81-1.15] | 1.04 [0.85-1.27] | 0.92 [0.71-1.50] | 1.30 [1.26-1.53] | 0.82 [0.73-0.97] |
| Ti min, s | **PS** | **S** | 0.76 [0.67-0.98] | 0.86 [0.80-1.13] | 0.92 [0.70-1.11] | 1.00 [0.86-1.07] | 0.79 [0.73-0.93] | 0.90 [0.70-1.26] | 0.93  [0.72-1.08] |
|  |  | **F** | 0.78 [0.69-0.94] | 0.76 [0.61-0.89] | 0.78 [0.62-0.90] | 0.90 [0.63-1.12] | 0.73 [0.69-1.19] | 1.13 [0.98-1.25] | 0.69 [0.60-0.90] |
|  | **PS+4** | **S** | 0.77 [0.77-0.93] | 0.89 [0.72-0.97] | 0.96 [0.74-1.52] | 0.93 [0.78-1.11] | 0.93 [0.79-1.09] | 0.87 [0.69-1.21] | 0.89 [0.77-1.10] |
|  |  | **F** | 0.74 [0.60-0.89] | 0.72 [0.60-0.84] | 0.77 [0.61-0.87] | 0.88 [0.71-1.01] | 0.71 [0.60-1.00] | 0.96 [0.77-1.29] | 0.67 [0.56-0.83] |
|  | **PS**  **-4** | **S** | 0.77 [0.67-0.89] | 0.84 [0.67-0.97] | 0.93 [0.81-1.05] | 0.90 [0.77-1.11] | 0.76 [0.72-0.95] | 0.85 [0.73-1.10] | 0.93 [0.79-1.11] |
|  |  | **F** | 0.74 [0.60-0.91] | 0.74 [0.61-0.90] | 0.73 [0.62-0.96] | 0.87 [0.67-1.03] | 0.82 [0.67-1.45] | 1.18 [1.08-1.25] | 0.71 [0.60-0.86] |
| Data presented as medians [interquartile range]. Differences within the group - Friedman test.  **Abbreviations:** S: Success; F: Failure; PS: Preset Pressure Support Level above positive end-expiratory pressure; PS+4: Preset Pressure Support Level above positive end-expiratory pressure plus 4 cmH_2_O; PS-4: Preset Pressure Support Level above positive end-expiratory pressure minus 4 cmH_2_O; VTe: exhaled tidal volume; IBW: ideal body weight; PIF: peak inspiratory flow; RR: respiratory rate; Ti: inspiratory time; MV: minute ventilation.  * p-value < 0.05, comparison within NIV success and NIV failure group during PS increase and decrease;  ** p-value < 0.01, comparison within NIV success and NIV failure group during PS increase and decrease;  § p-value < 0.001, comparison within NIV success and NIV failure group during PS increase and decrease. | | | | | | | | | |

**NIV failure prediction by the HACOR score**

The HACOR score was quite high at the time of initiation of NIV in all patients, and differed significantly between NIV success and NIV failure groups on Day 1, 3, 5, and 10 (**Table S2)**, although confidence intervals overlapped, unlike the ROX index (**Fig. S2**).

**Table S2. Changes in the HACOR score in NIV success and NIV failure groups during 14 days of NIV**

|  |  | **Day 1** | **Day 3** | **Day 5** | **Day 7** | **Day 10** | **Day 14** | **Last NIV day** |
| --- | --- | --- | --- | --- | --- | --- | --- | --- |
| HACOR score, points | **S** | 5.0** [0.0-6.0] | 4.0^§^ [0.0-5.0] | 4.5* [0.8-6.0] | 4.0 [0.0-6.0] | 4.0* [3.0-5.0] | 3.0 [0.5-4.8] | 0.0^§^ [0.0-3.0] |
|  | **F** | 6.0 [5.0-6.0] | 6.0 [6.0-7.0] | 6.0 [5.8-6.0] | 6.0 [4.0-6.0] | 6.0 [6.0-7.0] | 6.0 [4.0-6.0] | 6.0 [5.5-7.0] |
| Data presented as medians [interquartile range] or n (%) where appropriate. Differences between groups Mann-Whitney U-test.  **Abbreviations:** S: Success; F: Failure.  * p-value < 0.05, comparison between NIV success and NIV failure groups;  ** p-value < 0.01, comparison between NIV success and NIV failure groups;  § p-value < 0.001, comparison between NIV success and NIV failure groups. | | | | | | | | |

**
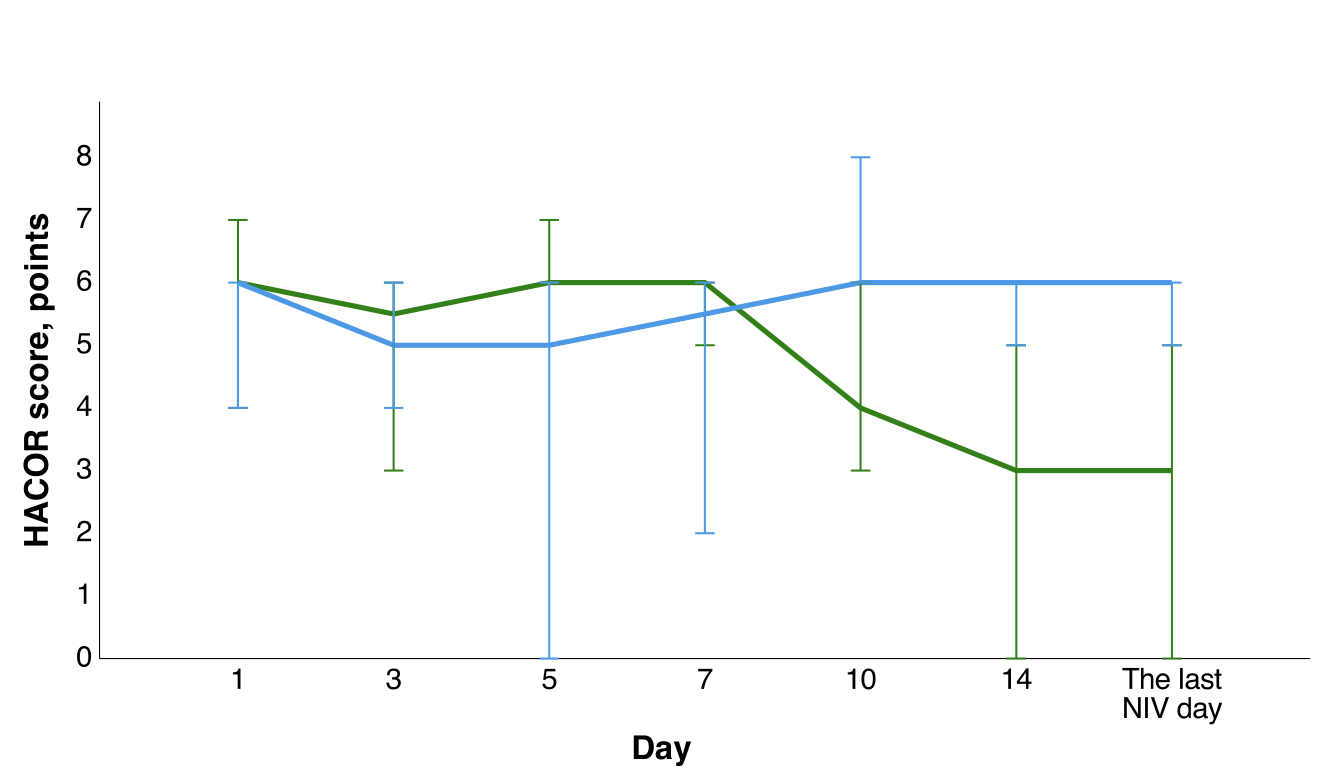
**

**Figure S2. Changes in the HACOR score in NIV success and NIV failure groups during 14 days.**

Data on NIV success (green) and NIV failure (blue) are presented as medians and 95% confidence intervals. The x-axis represents days after initiation of non-invasive ventilation.

The HACOR score > 5.5 after 48 hours of NIV can serve as a prognostic tool for NIV failure in moderate-to-severe COVID-19-associated ARDS (Se 83%, Sp 83%, AUROC 0.89 (0.81-0.97), p<0.001).


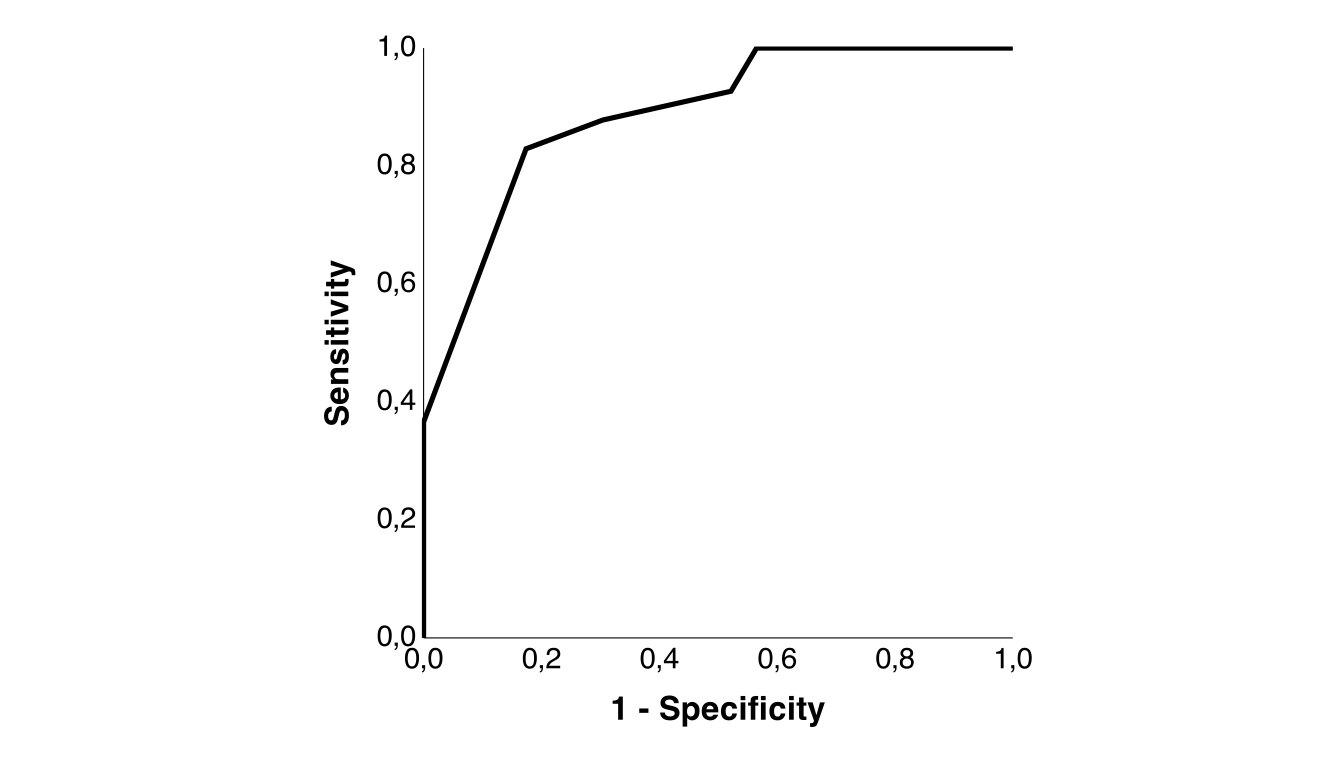


**Figure S3. Prediction of NIV failure by HACOR score after 48 hours of NIV (ROC curve)**
